# Individual Differences in Aesthetic Ability: The Case for an Aesthetic Quotient

**DOI:** 10.3389/fpsyg.2016.00750

**Published:** 2016-05-19

**Authors:** Nils Myszkowski, Franck Zenasni

**Affiliations:** ^1^Department of Psychology, Pace UniversityNew York, NY, USA; ^2^Laboratoire Adaptations Travail-Individu, Université Paris Descartes - Sorbonne Paris CitéParis, France

**Keywords:** aesthetic judgment, aesthetic appreciation, creativity, empirical aesthetics, aesthetic preference

Although the reasons individuals have specific stable aesthetic preferences—for example, for abstract art or for classical music—are often studied (e.g., Furnham and Walker, 2001), there is a growing stream of research (e.g., Nodine et al., 1993; Chamorro-Premuzic and Furnham, 2004; Axelsson, 2007; Kozbelt and Seeley, 2007; Silvia, 2007; Myszkowski et al., 2014) that is interested in the various abilities involved when evaluating art: Are we all equally “armed” to process aesthetic stimuli?

Our aim in this paper is to propose a new direction for this stream of research. While a typical approach to the study of aesthetic ability consists in measuring single facets, notably aesthetic sensitivity (e.g., Myszkowski et al., 2014), we propose a multi-content approach. More specifically, mirroring the “g-to-IQ” shift in intelligence measurement, we want to propose a “T-to-AQ” shift from single-content measures of “good taste” (“T”) to comprehensive assessments of an “Aesthetic Quotient” (AQ), which would include other facets of aesthetic ability—like artistic knowledge, sensitivity to complexity and aesthetic empathy. Rather that questioning the *existence* of an AQ, we argue its *usefulness*, notably in predicting creative potential and achievement.

## A central “T” construct

What we propose as a central construct to aesthetic ability has gone by many names—“Aesthetic Judgment” (Meier, 1940; Graves, 1948), “Aesthetic Perception” (Meier, 1963) or “Aesthetic Sensitivity” (Götz, 1985)—but it is commonly referred to as “T” for “Taste” (Eysenck, 1983; Myszkowski et al., 2014), which embraces the most clearly its definition: It is the ability to respond to aesthetic stimuli in agreement with ≪external standards≫ (Child, 1964). In other words, “T” is the ability to judge “well”—which, practically speaking, means “consensus-like” and/or “expert-like” (Myszkowski et al., 2014).

Although the letter “T” refers to Taste, it is important to note that “T” actually does not refer to personal taste (or personal preferences), but rather to the concept of “good taste”—“good” referring to relevant, appropriate or correct—as we commonly use it to describe individual characteristics. “T” is a specific ability involved in aesthetic experience (Myszkowski et al., 2014), which is a broader concept that refers to the entire activity relative to the processing of aesthetic stimuli (Leder et al., 2004).

### Evidence for “T”

It is interesting to note here that such a letter-sobriquet—originally proposed by Eysenck (1940)—is a direct reference to the general factor of intelligence, *g*. While Eysenck's provocative personality probably played a role in doing such a comparison, the “T” factor was indeed the result of the extraction—using exploratory factor analysis—of a principal component of aesthetic personal preferences, a lot like *g* was the result of the extraction of a principal component of intelligence test scores. Individuals tend to agree on the preference for aesthetic stimuli in most visual domains (Eysenck, 1940)—thus, consensual preferences exist for most stimuli—and the judges who tend to agree with average judgments are the same individuals for a wide variety of visual domains (Eysenck, 1940, 1941a)—therefore, individuals who agree with consensual preferences do so in a wide variety kinds of stimuli. These results point to a factor of individual differences that is stable across visual domains, and that corresponds to the extent to which our judgments are in agreement with consensually established standards.

### Measuring “T”

But measuring “T” is a challenge: How do we know if an individual responds to stimuli in accordance to “external standards”? How can we define “external standards”? The way that “T” has been discovered by Eysenck points to a clear way of addressing this issue: We identify or build pairs (or triads) of stimuli, so that one is preferred (by consensual agreement and experts) over one (or two) deteriorated versions of it. The aim of this first step is to build pairs or triads of designs, which each include a design of higher “objective” aesthetic quality than the others. These pairs or triads are then used as items: The test takers are asked to indicate, for each pair or triad, which of the two (or three) designs they consider of better objective quality (Meier, 1940, 1963; Graves, 1948; Götz, 1985; Myszkowski et al., 2014). A better “T” score is attributed to individuals who are better able to effectively recognize the stimuli that are aesthetically superior.

## Integrating connected constructs

### The lack of a pure “T” measure

Among what were designed as pure “T” measures, the Design Judgment Test (Graves, 1948) and the Visual Aesthetic Sensitivity Test (Götz, 1985) have been, because of their psychometrical qualities, the most heavily used (Chamorro-Premuzic and Furnham, 2004; Furnham and Chamorro-Premuzic, 2004; Myszkowski et al., 2014; Summerfeldt et al., 2015). However, “T” measures are restricted by their content (Gear, 1986). More specifically, when building these measures, researchers have built items that are not representative of the *entire* visual domain, and we could point to many signs of this lack of representativeness—the stimuli of these tests are mostly black and white (or black, gray and white) paintings (Götz, 1985). Consequently, these measures have been severely criticized as not being representative of “T.” Gear (1986, pp. 563–564), for example, sarcastically described the Visual Aesthetic Sensitivity Test as “a test of the ability to discriminate between greater and lesser degrees of ‘harmony’ in monochromatic two-dimensional figures in the same way as the well-known German painter Götz, eight other well-known painters and the well-known British psychologist Professor H. J. Eysenck.”

“T” is to aesthetic ability what *g* is to mental ability: Central, but difficult—if not impossible—to measure with a single test. However, while intelligence researchers switched from appreciated attempts to build pure “g” measures (Raven, 1941)—which are still not measures of pure intelligence (Gignac, 2015)—to comprehensive multifactorial IQ test batteries (e.g., Wechsler, 2008), this hasn't been the case for aesthetic ability, which hasn't evolved from “T” to AQ.

### Other AQ components

While measures that were designed to measure purely “T” may have failed in completely isolating it, it can be argued that these few measures are not the only tools that are available to capture aesthetic ability. The concept of aesthetic ability is multifaceted, and the measure of aesthetic ability should thus probably not be restricted to making aesthetic judgments of monochromatic paintings.

There is a set of existing constructs and measures that could be used in a comprehensive AQ assessment. We could propose for example the sensitivity to complex stimuli—named “K” in reference to the use of K for complexity in mathematics (Eysenck, 1941b)—which is already investigated in the field of creativity research, through the Figure-Preference Test (Barron and Welsh, 1952), and largely used to in the study of the creative personality (Eysenck and Furnham, 1993). We could also consider as an AQ component art knowledge, currently measured with the Aesthetic Fluency scale (Smith and Smith, 2006; Silvia, 2007) or the Aesthetic Experience Questionnaire (Chatterjee et al., 2010). Additionally, measures of aesthetic empathy (Lifton, 1961; Madsen et al., 1993; McCrae, 2007; Silvia and Nusbaum, 2011), or of the diversity of exploration patterns (Nodine et al., 1993) could be useful additions to a more comprehensive study of aesthetic ability.

**Figure 1:**
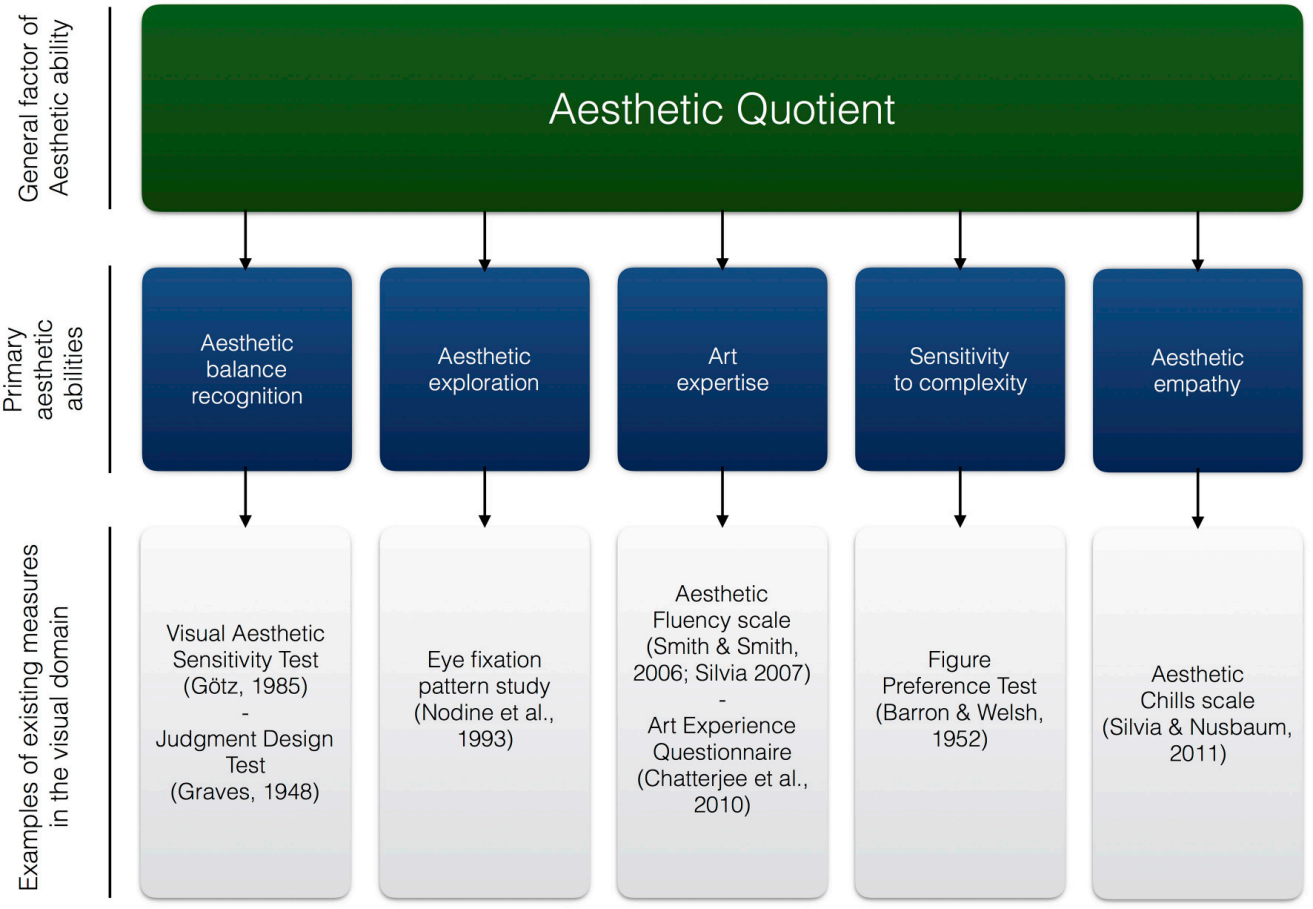
**Model for an Aesthetic Quotient approach of “T.”**.

Indeed, we here argue that various sources of individual differences—sensitivity to complexity, art knowledge, aesthetic empathy, attention, exploratory perception tendencies—constitute elements that make individuals more armed to process art and aesthetic stimuli, making them suitable candidates for inclusion in potential AQ assessment. However, this list is non-exhaustive and some of the existing measures (content, norms, scoring) would need to be updated before inclusion in a test battery. In Figure 1, we summarize our AQ approach. Because of their extensiveness in the visual domain, we propose example measures for each of the facets in this domain. We however think that similar measures should be found in other domains: For example, a pitch recognition test could be considered as measure of aesthetic balance recognition in the musical domain. Associated with the list of components that we propose, we could tentatively define AQ as the *global capacity to identify, explore, understand, seek stimulation in and respond to the elements, composition and meaning of art and aesthetic objects*.

## Why measure aesthetic ability?

### AQ is useful

The case has been previously made (e.g., Eysenck, 1988) that the *existence* of intelligence is not what is demonstrated by the discovery of *g*, but that such a discovery indicates a factor that is *useful* to explain and understand scores in mental ability tests, as well as to predict a variety of outcomes, like academic achievement. Making an analogous point for AQ, we acknowledge that the idea that aesthetic judgments can be of better or worse quality—and that, consequently, individuals capable of making better judgments have better taste—is a fascinating philosophical question, but we are not here positing that good taste *exists*: We are arguing, as it was suggested about “T” (Myszkowski et al., in press), that AQ is *useful* as a scientific concept, notably in the prediction and explanation of creativity.

While AQ concerns the “reception end” of aesthetic stimuli, it may be useful in the prediction of the “production end”: Creativity. Indeed, results suggest that the AQ components that we discussed earlier are helpful to predict a variety of outcomes that are creativity related. First, let us note that recent research has indicated that the Visual Aesthetic Sensitivity Test is a significant predictor of creative potential (Myszkowski et al., 2014). This suggests that individuals who are better equipped to judge aesthetic stimuli can probably use these tools to create. Additionally, the Visual Aesthetic Sensitivity Test and the Design Judgment Test were found to be related to creative personality traits like openness to aesthetics (Myszkowski et al., 2014). Besides, other AQ components that we proposed are also predictors of creativity. For example, aesthetic response intensity was found to be related to creative potential (Ziv and Keydar, 2009). Moreover, research indicates that artists have higher art expertise and process aesthetic stimuli differently (Kozbelt and Seeley, 2007). For example, photo professionals process photographic information more efficiently and prefer complex photos (Axelsson, 2007).

These results suggest that aesthetic sensitivity could be partly related to the development of creativity. To give examples of concrete applications, one could imagine that a designer's ability to create a highly ergonomic and efficient webpage design could be predicted by aesthetic ability. One could also propose training programs, using exploratory art perception activities, to enhance AQ among children or adults.

## Conclusion

We have here proposed the evolution of “T” toward a more comprehensive “AQ” approach and assessment of aesthetic ability. First, we have discussed the concept of “T” as a central factor of individual differences in the ability to judge aesthetic stimuli, and explained that results show that it is rather stable across a variety of categories of stimuli of a specific domain. We however noted that supposedly pure “T” measures have failed in fully encompassing aesthetic ability. We later proposed an evolution in the study of aesthetic ability, from attempting to purely measure “T” to a more comprehensive approach. We have proposed for this approach the term Aesthetic Quotient (AQ), as a reference to the “*g*-to-IQ” shift to comprehensive assessments of intelligence (e.g., Wechsler, 2008). We finally explained that psychology and empirical aesthetics researchers should probably stay clear of philosophical debates on the existence of aesthetic ability, and rather focus on the accumulating evidence on the usefulness of AQ components as predictors of creative potential and achievement.

## Author contributions

NM's contribution has consisted in conceiving the paper, drafting and revising the paper and the figure. FZ contributed substantially in the conception of the paper, and in critically improving the manuscript and enhancing its quality for publication.

## Funding

This article was supported by grant RFP-15-05 from the Imagination Institute (www.imagination-institute.org), funded by the John Templeton Foundation. The opinions expressed in this publication are those of the authors and do not necessarily reflect the view of the Imagination Institute or the John Templeton Foundation.

### Conflict of interest statement

The authors declare that the research was conducted in the absence of any commercial or financial relationships that could be construed as a potential conflict of interest.

## References

[B1] Axelsson Ö. (2007). Individual differences in preferences to photographs. Psychol. Aesthet. Creat. Arts 1, 61–72. 10.1037/1931-3896.1.2.61

[B2] Barron F. Welsh G. S. (1952). Artistic perception as a possible factor in personality style: its measurement by a figure preference test. J. Psychol. 33, 199–203. 10.1080/00223980.1952.9712830

[B3] Chamorro-Premuzic T. Furnham A. (2004). Art judgment: a measure related to both personality and intelligence? Imag. Cogn. Pers. 24, 3–24. 10.2190/U4LW-TH9X-80M3-NJ54

[B4] Chatterjee A. Widick P. Sternschein R. Smith W. B. Bromberger B. (2010). The assessment of art attributes. Empir. Stud. Arts 28, 207–222. 10.2190/EM.28.2.f

[B5] Child I. L. (1964). Observations on the meaning of some measures of esthetic sensitivity. J. Psychol. 57, 49–64. 10.1080/00223980.1964.9916671 14100130

[B6] Eysenck H. J. (1940). The general factor in aesthetic judgements. Br. J. Psychol. Gen. Sect. 31, 94–102. 10.1111/j.2044-8295.1940.tb00977.x

[B7] Eysenck H. J. (1941a). The empirical determination of an aesthetic formula. Psychol. Rev. 48, 83–92. 10.1037/h0062483

[B8] Eysenck H. J. (1941b). “Type” factors in aesthetic judgements. Br. J. Psychol. Gen. Sect. 31, 262–270. 10.1111/j.2044-8295.1941.tb00992.x

[B9] Eysenck H. J. (1983). A new measure of “good taste” in visual art. Leonardo 16, 229 10.2307/1574921

[B10] Eysenck H. J. (1988). The concept of “intelligence”: useful or useless? Intelligence 12, 1–16. 10.1016/j.diagmicrobio.2016.03.021 27160950

[B11] Eysenck H. J. Furnham A. (1993). Personality and the barron-welsh art scale. Percept. Mot. Skills 76, 837–838. 10.2466/pms.1993.76.3.837 8321595

[B12] Furnham A. Chamorro-Premuzic T. (2004). Personality, intelligence, and art. Pers. Individ. Diff. 36, 705–715. 10.1016/S0191-8869(03)00128-4

[B13] Furnham A. Walker J. (2001). Personality and judgements of abstract, pop art, and representational paintings. Eur. J. Pers. 15, 57–72. 10.1002/per.340

[B14] Gear J. (1986). Eysenck's visual aesthetic sensitivity test (VAST) as an example of the need for explicitness and awareness of context in empirical aesthetics. Poetics 15, 555–564. 10.1016/0304-422X(86)90011-2

[B15] Gignac G. E. (2015). Raven's is not a pure measure of general intelligence: Implications for g factor theory and the brief measurement of g. Intelligence 52, 71–79. 10.1111/j.1365-2788.2008.01045.x 18312310

[B16] Götz K. O. (1985). VAST: Visual Aesthetic Sensitivity Test, 4th Edn. Dusseldorf: Concept Verlag.

[B17] Graves M. E. (1948). Design Judgment Test. New York, NY: Psychological Corporation.

[B18] Kozbelt A. Seeley W. P. (2007). Integrating art historical, psychological, and neuroscientific explanations of artists' advantages in drawing and perception. Psychol. Aesthet. Creat. Arts 1, 80–90. 10.1037/1931-3896.1.2.80

[B19] Leder H. Belke B. Oeberst A. Augustin D. (2004). A model of aesthetic appreciation and aesthetic judgments. Br. J. Psychol. 95, 489–508. 10.1348/0007126042369811 15527534

[B20] Lifton W. M. (1961). The development of a music reaction test to measure affective and aesthetic sensitivity. J. Res. Music Educ. 9, 157–166. 10.2307/3344311

[B21] Madsen C. K. Brittin R. V. Capperella-Sheldon D. A. (1993). An empirical method for measuring the aesthetic experience to music. J. Res. Music Educ. 41, 57–69. 10.2307/3345480

[B22] McCrae R. R. (2007). Aesthetic chills as a universal marker of openness to experience. Motiv. Emotion 31, 5–11. 10.1007/s11031-007-9053-1

[B23] Meier N. C. (1940). The Meier Art Tests: I, Art Judgment. Iowa City, IA: Bureau of Educational Research and Service, University of Iowa.

[B24] Meier N. C. (1963). The Meier Art Tests: II, Aesthetic Perception. Iowa City, IA: Bureau of Educational Research and Service, University of Iowa.

[B25] Myszkowski N. Storme M. Zenasni F. (in press). Order in complexity: how hans eysenck brought differential psychology aesthetics together. Pers. Individ. Diff.

[B26] Myszkowski N. Storme M. Zenasni F. Lubart T. (2014). Is visual aesthetic sensitivity independent from intelligence, personality and creativity? Pers. Individ. Dif. 59, 16–20. 10.1016/j.paid.2013.10.021

[B27] Nodine C. F. Locher P. J. Krupinski E. A. (1993). The role of formal art training on perception and aesthetic judgment of art compositions. Leonardo 26, 219 10.2307/1575815

[B28] Raven J. C. (1941). Standardization of progressive matrices, 1938. Br. J. Med. Psychol. 19, 137–150. 10.1111/j.2044-8341.1941.tb00316.x

[B29] Silvia P. J. (2007). Knowledge-based assessment of expertise in the arts: Exploring aesthetic fluency. Psychol. Aesthet. Creat. Arts 1, 247–249. 10.1037/1931-3896.1.4.247

[B30] Silvia P. J. Nusbaum E. C. (2011). On personality and piloerection: Individual differences in aesthetic chills and other unusual aesthetic experiences. Psychol. Aesthet. Creat. Arts 5, 208–214. 10.1037/a0021914

[B31] Smith L. F. Smith J. K. (2006). The nature and growth of aesthetic fluency, in New Directions in Aesthetics, Creativity and the Arts eds Locher P. Martindale C. Dorfman L. (Amityville, NY: Baywood Publishing Co), 47–58.

[B32] Summerfeldt L. J. Gilbert S. J. Reynolds M. (2015). Incompleteness, aesthetic sensitivity, and the obsessive-compulsive need for symmetry. J. Behav. Ther. Exp. Psychiatry 49, 141–149. 10.1016/j.jbtep.2015.03.006 25823552

[B33] Wechsler D. (2008). Wechsler Adult Intelligence Scale, 4th Ed (WAIS–IV). San Antonio, TX: NCS Pearson.

[B34] Ziv N. Keydar E. (2009). The relationship between creative potential, aesthetic response to music, and musical preferences. Creat. Res. J. 21, 125–133. 10.1080/10400410802633764

